# Restless legs syndrome and sleep quality in children with migraine and tension-type headache

**DOI:** 10.7717/peerj.20925

**Published:** 2026-03-16

**Authors:** Hicran Altın, Fatih Ay, Muhammet Kutluk

**Affiliations:** 1 Department of Child Health and Diseases/Antalya Health Research Center, University of Health Sciences, Antalya, Turkey; 2 Department of Pediatric Neurology/Antalya Health Research Center, University of Health Sciences, Antalya, Turkey

**Keywords:** Migraine disorders, Tension-type headache, Restless legs syndrome, Sleep quality, Sleep wake disorders, Pediatrics

## Abstract

**Background:**

Headache disorders and sleep disturbances are common in children, with migraine and tension-type headache (TTH) being the most prevalent primary headache subtypes. Restless legs syndrome (RLS) is more frequently reported in children with migraine and may exacerbate sleep disturbances and related cognitive and quality-of-life impairments via shared neurobiological mechanisms; its prevalence across pediatric headache subtypes and its association with sleep quality remain insufficiently characterized. Therefore, this study aimed to assess the prevalence of RLS in children diagnosed with migraine or TTH and to examine its relationship with sleep quality.

**Methods:**

In this cross-sectional study, 148 children with primary headaches (99 migraine, 49 TTH) and 148 age- and sex-matched healthy controls were included. The diagnosis of RLS was established using the pediatric criteria of the International Restless Legs Syndrome Study Group. Sleep quality was assessed using the Pittsburgh Sleep Quality Index (PSQI). Group comparisons were performed to evaluate differences in RLS prevalence among headache subtypes and controls using chi-square tests, while group comparisons of continuous variables were performed using nonparametric tests. Spearman correlation was used to assess the relationship between RLS and sleep quality. In addition, mediation analysis was conducted to explore the role of RLS presence in the association between migraine and sleep quality.

**Results:**

RLS was detected in 20.9% of participants with primary headaches and in 8.8% of healthy controls, with a statistically significant difference between the groups (*p* = 0.003). Children with primary headaches had significantly higher PSQI scores compared to healthy controls (*p* = 0.002). Subgroup analysis demonstrated a significant difference in RLS prevalence among migraine, TTH, and control groups (*p* = 0.001), with RLS being most frequent in children with migraine (25.3%), followed by those with TTH (12.2%) and healthy controls (8.8%). Children with co-occurring migraine and RLS exhibited significantly poorer sleep quality compared with those with migraine alone (*p* < 0.001) In addition, a statistically significant positive correlation was observed between RLS symptom severity and impaired sleep quality (*r* = 0.571, *p* < 0.001). Mediation analysis indicated that RLS presence partially explained the association between migraine and impaired sleep quality (34.2% mediation, *p* = 0.008).

**Conclusion:**

RLS was observed significantly more frequently among children with primary headaches, particularly migraine, and was significantly associated with poorer sleep quality. RLS may act as a contributing factor in the relationship between migraine and sleep impairment. These findings highlight the importance of evaluating RLS and sleep disturbances during the clinical assessment of pediatric patients with recurrent headache.

## Introduction

Headache disorders and sleep disturbances are among the most frequent neurological complaints during childhood and adolescence, negatively impacting cognitive development, emotional regulation, and overall quality of life (Karaaslan et al., 2023; Sun et al., 2021). Migraine and tension-type headache (TTH) are the most common primary headache types observed in pediatric populations, with migraine generally associated with greater disability compared to TTH (Faedda et al., 2016; Seidel et al., 2012; Sevindik et al., 2017).

Although both are categorized as primary headache disorders, migraine and TTH differ in their underlying mechanisms and clinical characteristics. Migraine presents as recurrent, unilateral, pulsating pain of moderate to severe intensity, often accompanied by nausea, vomiting, photophobia, or phonophobia. TTH, on the other hand, is characterized by bilateral, pressing pain of mild to moderate intensity that is not usually worsened by physical exertion or associated with sensory disturbances. These distinctions are clinically important, as they may reflect different neurobiological pathways and influence related symptoms such as sleep disruption (Headache Classification Committee of the International Headache Society, 2018).

Restless legs syndrome (RLS), a sensorimotor disorder characterized by an uncontrollable urge to move the legs during periods of rest, has been shown to significantly impair both sleep quality and daily (Allen et al., 2014; Picchietti et al., 2013). The prevalence of RLS among healthy children has been reported to range between 2% and 10%, depending on diagnostic criteria and population characteristics (Picchietti & Picchietti, 2008; Silva et al., 2014). Several studies have reported a higher prevalence of RLS in children with migraine compared to healthy controls (Seidel et al., 2012; Sevindik et al., 2017; Valente et al., 2017).

Both migraine and RLS independently contribute to sleep disturbances, and their coexistence may further exacerbate sleep-related problems (DelRosso, Bruni & Ferri, 2020; Van Oosterhout et al., 2016; Wang et al., 2019; Wood, 2016). Given the essential role of sleep in physical growth, brain maturation, emotional regulation, and psychosocial development during childhood (Agostini & Centofanti, 2024; Owens, 2008), disruption of sleep during critical developmental periods may result in long-term negative effects on academic performance, emotional well-being, and overall functioning (Cortese et al., 2009; d’Onofrio et al., 2011).

Previous studies have examined migraine, tension-type headache, RLS, and sleep problems separately. Only a few have compared how often and how severely RLS occurs, and how it affects sleep quality, in different types of primary headaches in children. Addressing this gap, the present study aimed to assess the prevalence and severity of RLS in children diagnosed with migraine or TTH and to compare these findings with those of age- and sex-matched healthy controls. Additionally, the study sought to examine the relationship between RLS symptom severity and sleep quality, thereby providing a more comprehensive understanding of the overlapping burden of primary headache disorders, RLS, and sleep disturbances in children.

## Materials & Methods

### Study design and setting

This study employed a cross-sectional study design conducted at the Pediatric Neurology Department of Antalya Health Research Center between September 1, 2021 and May 1, 2022. Written informed consent was obtained from all participating parents in the study. The research adhered to the ethical guidelines outlined in the in the Declaration of Helsinki. The Clinical Research Ethics Committee of Antalya Training and Research Hospital, University of Health Sciences approved the study protocol prior to study initiation (Ethical Application Ref: 2/20, Date: 20/01/2022). Strengthening the Reporting of Observational Studies in Epidemiology (STROBE) guidelines were followed in this study.

### Participant selection

Children aged 8 to 18 years who were followed in the pediatric neurology clinic with a diagnosis of migraine or TTH, established according to the International Classification of Headache Disorders, 3rd edition (ICHD-3) (Zhang et al., 2016), were included in the case group. During the study period, both cases and controls were evaluated for the presence of RLS using the International Restless Legs Syndrome Study Group (IRLSSG) pediatric diagnostic criteria. The RLS diagnosis was established based on current clinical evaluation at the time of data collection, without applying a time-to-diagnosis criterion.

The control group included healthy children without chronic medical conditions who attended the same hospital’s general pediatric clinic during the same period. Controls were frequency-matched to cases by age and sex to ensure comparable demographic distributions between groups rather than individual (one-to-one) matching. All control participants were initially evaluated by a general pediatrician and were subsequently assessed by a pediatric neurologist specifically.

Children with medical conditions known to be associated with secondary RLS, including iron deficiency anemia, vitamin B12/folate or vitamin D deficiency, chronic kidney disease, type 2 diabetes mellitus, ADHD, multiple sclerosis, and systemic inflammatory or endocrine disorders, were excluded. Additionally, individuals with peripheral neuropathy, previously diagnosed sleep disorders, or the use of medications affecting sleep or RLS symptoms were not included. Patients with secondary headache causes such as hypertension, ocular diseases, infections, or intracranial pathology on neuroimaging were also excluded.

**Figure 1:**
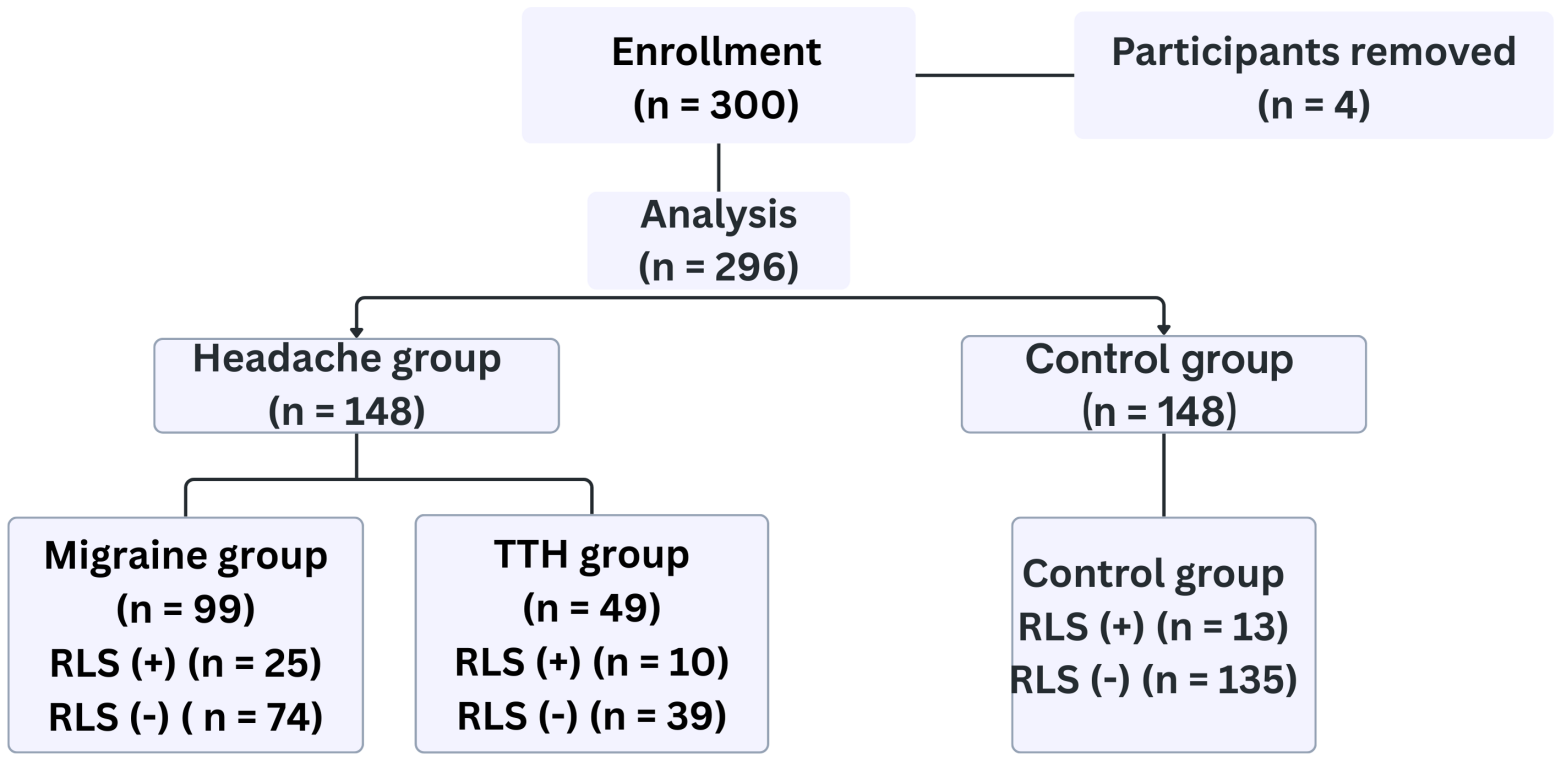
Participant distribution by headache subtype and restless legs syndrome (RLS) status. TTH, Tension-Type Headache; RLS, Restless Legs Syndrome.

The allocation of participants into study groups and their RLS status are illustrated in Fig. 1.

### Assessment of restless legs syndrome

The diagnosis of RLS was established using the consensus criteria of the IRLSSG (Allen et al., 2014). RLS is a clinical diagnosis and does not require polysomnography for confirmation. According to the IRLSSG, the diagnosis is based on the fulfillment of essential clinical criteria derived from patient-reported symptoms. Polysomnography is not included in the diagnostic criteria and is generally reserved for atypical cases or for the assessment of periodic limb movements during sleep (Gossard et al., 2021).

For younger participants, a pediatric-adapted version of the IRLSSG questionnaire was employed with assistance from parents or guardians to ensure diagnostic reliability (Picchietti et al., 2013). In all figures and tables, RLS (+) denotes participants who met the diagnostic criteria for restless legs syndrome according to the IRLSSG, whereas RLS (−) denotes those without the condition. Among participants diagnosed with RLS, symptom severity was systematically evaluated using the standardized IRLSSG Rating Scale. This standardized scale comprises 10 items assessing the severity and frequency of RLS symptoms, each rated on a five-point Likert scale from 0 to 4. The total score ranges from 0 to 40, with higher scores indicating greater symptom severity. Based on the total score, RLS severity was classified as mild (0–10), moderate (11–20), severe (21–30), and very severe (31–40) (Chen et al., 2010).

### Assessment of sleep quality

Sleep quality was assessed utilizing the Pittsburgh Sleep Quality Index (PSQI), a validated self-report instrument designed to measure sleep disturbances over a one-month period (Buysse et al., 1989). The PSQI was completed by the participants themselves, with parental assistance provided if necessary. A global PSQI score greater than 5 was considered indicative of clinically significant sleep disturbance.

### Sample size and statistical analysis

The calculation was based on previously published pediatric data reporting a 22% prevalence of RLS in the headache group and approximately 5–8% in healthy controls (Seidel et al., 2012). Using these parameters (two-sided *α* = 0.05, power = 80%, case: control = 1:1), the least detectable odds ratio was 3.24, yielding a minimum required sample size of 116 cases and 116 controls. To ensure adequate statistical power for subgroup analyses (migraine, tension-type headache, and healthy controls) and to account for potential exclusions and attrition, the total sample was expanded. A total of 300 children were initially enrolled. However, four participants withdrew before completing the study protocol. Accordingly, the final sample comprised 296 participants, 148 in the headache group and 148 in the control group.

All statistical analyses were performed using IBM SPSS Statistics for Windows, version 27.0 (IBM Corp., Armonk, NY, USA). The normality of continuous variables was assessed using the Shapiro–Wilk test, and none showed a normal distribution. Therefore, continuous variables were summarized as median and interquartile range (IQR), and categorical variables were presented as frequencies and percentages. Group comparisons for categorical variables were performed using Pearson’s chi-square test. When the assumptions for the chi-square test were not fully met (*i.e.,* when expected cell counts were small), the continuity-corrected chi-square test was applied. Comparisons between two groups were performed using the Mann–Whitney U test for continuous variables. When comparisons involved more than two groups, the Kruskal–Wallis test was employed, followed by Bonferroni-corrected post hoc pairwise comparisons when significant differences were detected. Relationships between RLS severity and PSQI scores were assessed using Spearman’s correlation coefficient (r).

To further investigate the interrelationship between migraine, RLS, and sleep quality, a mediation analysis was performed. Migraine status was entered as the independent variable, presence of RLS (yes/no) as the mediator, and PSQI score as the dependent variable. Mediation analyses were conducted using Jamovi software (version 2.7).

A two-tailed *p*-value < 0.05 was considered statistically significant.

## Results

### Participant characteristics

A total of 296 children were included in the study, comprising 148 in the headache group (99 with migraine and 49 with TTH and 148 in the healthy control group. The median age was 13.5 years (IQR: 10.55–15.85) in the headache group and 12.95 years (IQR: 10.2–15.3) in the control group, and no statistically significant difference was observed based on the Mann–Whitney U test (*U* = 10,298.5, *p* = 0.375). The sex distribution was also comparable between groups, as determined by the Pearson chi-square test (*χ*^2^ = 0.054, *p* = 0.816). No significant differences were found between the groups in terms of hemoglobin, ferritin, free T4, TSH, vitamin B12, folate, and vitamin D levels, according to the Mann–Whitney U test (all *p* > 0.05). RLS was identified in 31 children (20.9%) in the headache group and 13 children (8.8%) in the healthy control group, as determined by the Pearson chi-square test (*p* = 0.003) The headache group had significantly higher PSQI scores (4.0 (IQR: 2–5)) than control group (2.5 (IQR: 1–4)). These findings are presented in Table 1.

**Table 1:** Demographic and laboratory characteristics of the study population.

**Variables**	**Headache Group (+)** **n=148**	**Control Group** **n=148**	***χ^2^ /U*** ** values**	***P*** **-value**
Age (years), median (IQR)	13.5 (10.55–15.85)	12.95 (10.2–15.3)	*U* = 10,298.5	0.375^*^
Sex, n (%)			*χ*^2^= 0.054	0.816^**^
• Female	77 (52%)	75 (50.7%)		
• Male	71 (48%)	73 (49.3%)		
Hemoglobin (g/dL), median (IQR)	13.2 (12.75–13.95)	13.05 (12.6–13.7)	*U* = 9,887.0	0.174^*^
Ferritin (μ g/L), median (IQR)	27 (18–36)	28.5 (17.5–39.5)	*U* = 10,266.5	0.352^*^
Free T4 (ng/dL), median (IQR)	0.86 (0.78–0.96)	0.83 (0.77–0.89)	*U* = 9,566.5	0.060^*^
TSH (uIU/mL), median (IQR)	1.88 (1.26–2.49)	1.82 (1.33–2.59)	*U* = 10,762.0	0.797^*^
Vitamin B12 (ng/L), median (IQR)	247 (188.5–320)	271 (187.5–323)	*U* = 10,154.0	0.368^*^
Folic acid (μ g/L), median (IQR)	9.98 (8.06–12.5)	9.28 (7.39–11.59)	*U* = 9,609.0	0.068^*^
Vitamin D (μ g/L), median (IQR)	23.2 (19–26.4)	22.3 (15.6–28.1)	*U* = 10,859.0	0.899^*^
Presence of RLS, n (%)	31 (20.9%)	13 (8.8%)	*χ*^2^= 7.72	0.003^***^
Female, n (%)	13 (41.9%)	5 (38.5%)		
Male, n (%)	18 (58.1%)	8 (61.5%)		
RLS severity score (*n* = 43), median (IQR)	16 (13–21)	12 (10–16)	*U* = 136.5	0.093^*^
PSQI, median (IQR)	4 (2–5)	2.5 (1–4)	*U* = 8,693.0	0.002^*^

**Notes.**

RLS restless legs syndrome TSH thyroid stimulating hormone IQR Interquartile Range Q1-Q3 PSQI Pittsburgh Sleep Quality Index

** Pearson’s chi-square test.

∗ Mann–Whitney U test.

*** Continuity correction chi square test.

In the subgroup analysis, RLS was identified in 25 of 99 children with migraine (25.3%) and in six of 49 children with tension-type headache (12.2%), while 13 of 148 healthy controls (8.8%) also met the criteria for RLS. The difference in RLS prevalence among the three groups was statistically significant (*χ*^2^ = 13.03, *p* = 0.001). Post-hoc pairwise comparisons indicated that the prevalence of RLS was significantly higher in the migraine group compared with healthy controls (*p* = 0.001), whereas no significant difference was observed between the TTH and control groups (*p* = 0.576) or between the TTH and migraine groups (*p* = 0.106). RLS severity scores also differed significantly across groups (*H* = 6.571, *p* = 0.037), with post-hoc tests showing higher scores in the migraine group compared with healthy controls (*p* = 0.035). Regarding sleep quality, PSQI scores were significantly different among the three groups (*H* = 12.085, *p* = 0.002). Significant pairwise differences were observed between the healthy control and migraine groups (*p* = 0.001). No significant differences were found between the tension-type headache group and either the migraine or control groups (Table 2).

**Table 2:** Comparison of restless legs syndrome presence, severity, and PSQI score among children with tension-type headache, migraine, and healthy controls.

	**Healthy controls** ***n* = 148**	**TTH** ***n* = 49**	**Migraine** ***n* = 99**	***χ^2^/H*** ** values**	***P*** **-value**
**RLS, n (%)** Yes No	13 (8.8) 135 (91.2)	6 (12.2) 43 (87.8)	25 (25.3) 74 (74.7)	*χ^2^= 13.03*	0.001^*^
**RLS severity score** Median (IQR)	12 (10–16)	13.5 (11–16)	17 (15–22)	*H* = 6.571	0,037^**^
**PSQI, median (IQR)** Median (IQR)	2.5 (1–4)	3 (1–4)	4 (2–6)	*H* = 12.085	0,002^**^

**Notes.**

RLS restless legs syndrome TTH tension-type headache IQR interquartile range, Q1–Q3

* Pearson’s chi-square test.

** Kruskal–Wallis test.

Bonferroni correction was applied for multiple comparisons.

Significant pairwise differences for RLS prevalence Migraine *vs* Healthy Controls, *p* = 0.001; Healthy *vs* TTH, *p* = 0.576; TTH *vs* Migraine, *p* = 0.106.

Significant pairwise differences for RLS severity: Healthy *vs* Migraine, *p* = 0.035; Healthy *vs* TTH, *p* = 0.757; TTH *vs* Migraine, *p* = 0.053.

Significant pairwise differences for PSQI score: Healthy *vs* Migraine, *p* = 0.001; Healthy *vs* TTH, *p* = 0.264; TTH *vs* Migraine, *p* = 0.102.

Sleep quality was found to be significantly poorer among children with RLS. In the migraine group, participants with RLS had significantly higher PSQI scores (7.0 (IQR: 5–9)) than those without RLS (3.0 (IQR: 1–4)), as indicated by the Mann–Whitney U test (*U* = 300.5, *p* < 0.001). In the control group, RLS-positive participants also showed significantly higher PSQI scores (4.0 (IQR: 4–5)) compared to RLS-negative participants (2.0 (IQR: 1–4)) (*U* = 428.0, *p* = 0.002). No statistically significant difference in PSQI scores was observed within the TTH group (*U* = 117.0, *p* = 0.645). These findings are presented in Table 3.

**Table 3:** Comparison of restless legs syndrome presence, severity, and PSQI score among children with tension-type headache, migraine, and healthy controls.

**Groups**	**PSQI Median (IQR)**	***U*** ** values**	***P*** **-value**
**Control Group –RLS (–),***n* = 135 **Control Group –RLS (+),***n* = 13	2 (1–4) 4 (4–5)	428.0	0.002^*^
**TTH–RLS (–),***n* = 43 **TTH –RLS (+),***n* = 6	3 (1–4) 3 (3–4)	117.0	0.645^*^
**Migraine –RLS (–),***n* = 74 **Migraine –RLS (+),***n* = 25	3 (1–4) 7 (5–9)	300.5	<0.001^*^

**Notes.**

RLS (+) participants diagnosed with restless legs syndrome according to the International Restless Legs Syndrome Study Group (IRLSSG) criteria RLS(–) participants without restless legs syndrome PSQI Pittsburgh Sleep Quality Index TTH tension-type headache IQR interquartile range, Q1-Q3

* Mann–Whitney U test.

**Figure 2:**
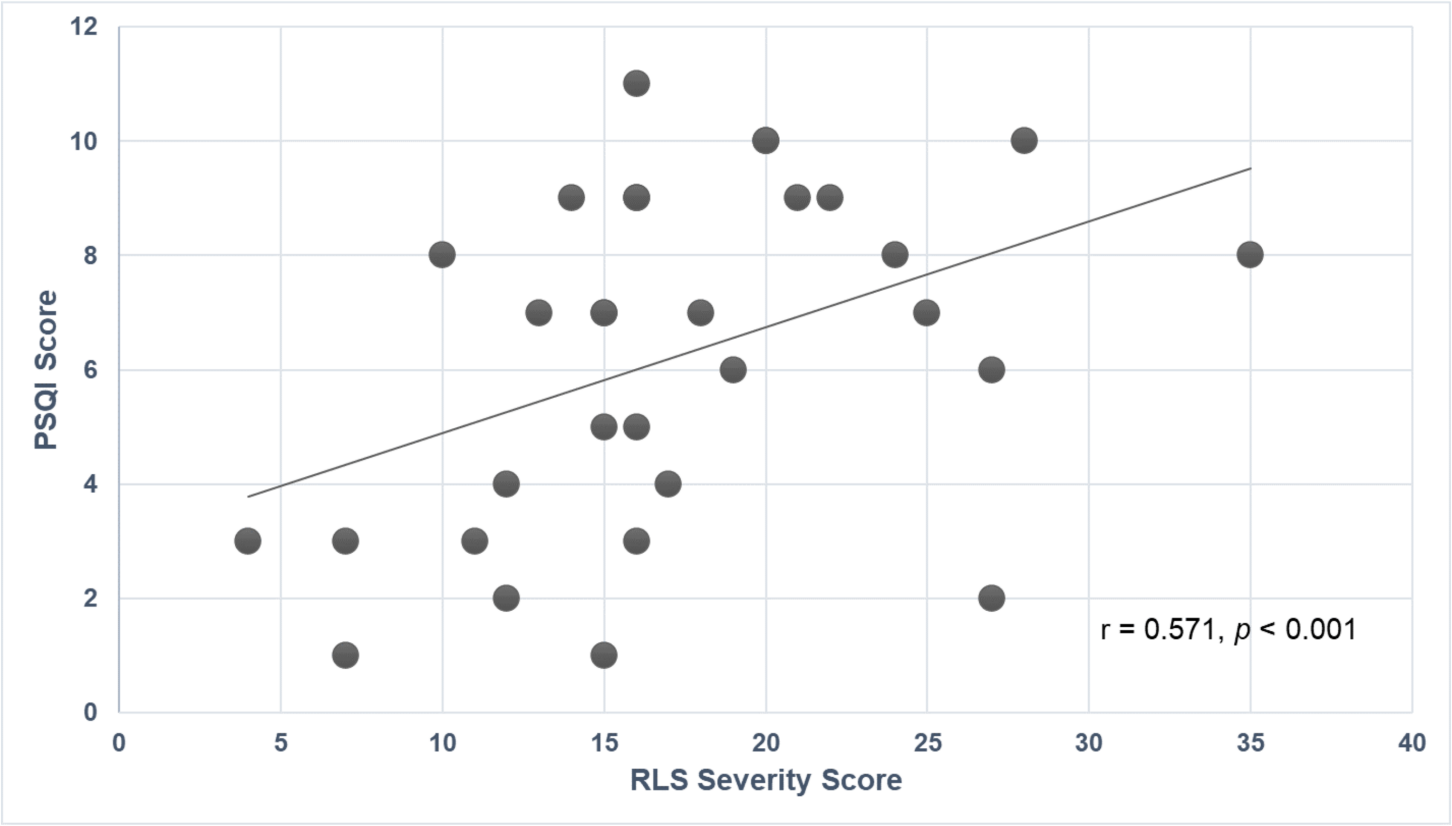
Correlation between RLS severity and PSQI scores in participants with RLS. RLS, Restless Legs Syndrome; PSQI, Pittsburgh Sleep Quality Index.

Among participants diagnosed with RLS, a moderate and statistically significant positive correlation was observed between RLS severity scores and global PSQI scores, as determined by Spearman’s rank correlation test (*r* = 0.571, *p* < 0.001) (Fig. 2).

Mediation analysis demonstrated a statistically significant indirect effect of migraine on sleep quality through the presence of RLS (indirect effect = 0.413, 95% CI [0.145–0.745], *p* = 0.008), accounting for 34.2% of the total effect. The direct effect of migraine on PSQI score remained significant after adjustment for RLS presence (direct effect = 0.795, 95% CI [0.239–1.358], *p* = 0.005), indicating partial mediation (Table 4). The results indicated that RLS presence statistically explained part of the association between migraine and impaired sleep quality, whereas migraine remained directly associated with sleep quality independent of RLS.

**Table 4:** Mediation analysis examining the role of RLS presence in the association between migraine and sleep quality.

a. Mediation Estimates						
Effect	Estimate	SE	95% CI	*Z*	*p*	% Mediation
Indirect effect (a × b)	0.413	0.156	0.145–0.745	2.64	0.008	34.2
Direct effect (c)	0.795	0.284	0.239–1.358	2.80	0.005	65.8
Total effect (c + a × b)	1.208	0.328	0.564–1.865	3.69	<0.001	100.0

b. Path Estimates					
Path	Estimate	SE	95% CI	*Z*	*p*
Migraine → RLS (a)	0.156	0.046	0.063–0.244	3.39	<0.001
RLS → PSQI (b)	2.645	0.433	1.823–3.470	6.10	<0.001
Migraine → PSQI (c)	0.795	0.284	0.239–1.358	2.80	0.005

**Notes.**

SE Standard Error PSQI Pittsburgh Sleep Quality Index RLS restless legs syndrome CI Confidence Interval

## Discussion

In this cross-sectional study, the prevalence and severity of RLS were evaluated in children diagnosed with primary headache disorders, including migraine and TTH, and compared with those of age- and sex-matched healthy controls. RLS was detected in 20.9% of children with headaches and in 8.8% of controls. Subgroup analysis showed that 25.3% of children with migraine and 12.2% of those with TTH fulfilled the diagnostic criteria for RLS, whereas the prevalence among controls remained at 8.8%. Although a slight increase in RLS prevalence was observed in the TTH group compared to controls, this difference did not reach statistical significance. Importantly, further mediation analysis demonstrated that the presence of RLS accounted for a substantial proportion of the association between migraine and impaired sleep quality, suggesting that the increased prevalence of RLS in migraine is clinically meaningful rather than incidental.

Consistent with previous studies (Chen et al., 2010; d’Onofrio et al., 2008; d’Onofrio et al., 2011), the present study found that the frequency of RLS was slightly higher in the TTH group compared with the control group; however, this difference did not reach statistical significance. In contrast, Yang et al. (2015) in a large nationwide, population-based cohort study, reported that TTH was significantly associated with an increased risk of developing RLS (HR = 1.57, 95% CI [1.22–2.02]), particularly among individuals aged 20–39 years, who exhibited an approximately 2.6-fold higher risk compared with those without TTH. The inconsistency between the present findings and previous reports may be partly attributable to differences in study populations, as prior studies predominantly involved adult cohorts, whereas the current investigation was conducted in a pediatric population. Accordingly, age-related variations in neurobiological mechanisms, disease duration, and comorbidity profiles may have contributed to the absence of a statistically significant association between TTH and RLS in children.

In contrast, a significantly higher prevalence and greater severity of RLS were observed in the migraine subgroup, indicating a more specific association between migraine and RLS than with primary headache disorders in general. These results are consistent with previous studies reporting a higher frequency of RLS in pediatric patients with migraine relative to healthy peers, thereby supporting the hypothesis of a shared pathophysiological mechanism (Seidel et al., 2012; Sevindik et al., 2017; Valente et al., 2017; Wang et al., 2019).

In the present study, the prevalence of RLS among healthy children was found to be 8.8%. This finding is consistent with previous reports. For instance, Seidel et al. (2012) reported an RLS prevalence of 6.5% among healthy adolescents, while Silva et al. (2014) documented rates ranging from 2% to 8% in pediatric populations. Moreover, a recent systematic review and modelling study conducted by Song et al. (2024) which included data from 23 countries, estimated the global prevalence of RLS in adults to be approximately 7.12%. Collectively, these findings suggest that while RLS can occur in healthy individuals, its prevalence appears to be lower compared to individuals diagnosed with primary headache disorders, particularly migraine.

Our findings demonstrated that children with primary headaches, particularly migraine, may experience RLS more frequently than their healthy controls. In contrast, the frequency of RLS in the tension-type headache group was similar to that of healthy controls, and the difference between them did not reach significance. These results are consistent with previous studies in both adult and pediatric populations showing a significant association between migraine and RLS (Chen et al., 2010; d’Onofrio et al., 2008; Schurks et al., 2012; Sevindik et al., 2017; Zanigni et al., 2014).

Chen et al. (2010) proposed that shared dopaminergic dysfunction within the A11 nucleus may underlie this comorbidity, whereas d’Onofrio et al. (2008) suggested that migraine could act as a risk factor for RLS, reflecting overlapping neurobiological mechanisms. Similarly, Sevindik et al. (2017) reported a higher prevalence of RLS among children with migraine and tension-type headache compared with healthy controls, emphasizing the clinical importance of screening for RLS in pediatric headache patients. These results collectively support the view that migraine, regardless of subtype, may serve as a clinical indicator of increased RLS risk in children.

Regarding symptom severity, children with migraine and comorbid RLS exhibited notably higher RLS symptom scores compared to controls. No significant difference in RLS severity was observed between the TTH group and controls, indicating that migraine not only increases RLS prevalence but also intensifies symptom burden. In alignment with the present findings, significantly higher RLS severity scores among migraine patients compared to healthy controls were reported by Van Oosterhout et al. (2016) (*p* = 0.036). A meta-analysis by Wang et al. (2019) similarly indicated that across all reviewed studies, RLS patients with migraine demonstrated higher severity scores compared to those without migraine, although statistical significance was consistently demonstrated only in one study. These observations suggest that migraine may not only predispose individuals to the development of RLS, but may also exacerbate the symptom burden, even in pediatric populations.

The shared pathophysiological basis of migraine and RLS has been proposed to involve mechanisms such as dopaminergic dysfunction, central iron deficiency, and neuroinflammatory activity (Schurks et al., 2014; Silvestri & DelRosso, 2021; Trenkwalder, Paulus & Walters, 2005). Support for this hypothesis has been provided by neuroimaging studies, including findings by Yang et al. (2019) who identified both disorder-specific and convergent gray matter volume (GMV) alterations in individuals with migraine and RLS. Among the regions implicated, the middle frontal gyrus consistently exhibited GMV differences across both conditions and was further associated with impaired sleep quality in comorbid cases. These structural alterations have been interpreted as neurobiological evidence of a potential shared etiological mechanism between migraine and RLS. In addition, increased RLS severity in pediatric migraine patients has been associated with vitamin D deficiency (Cederberg, Silvestri & Walters, 2023; Sun et al., 2021).

Sleep is considered essential for healthy growth, neurological development, emotional stability, and cognitive functioning during both childhood and adolescence (Agostini & Centofanti, 2024; Owens, 2008). In the current investigation, a statistically significant positive correlation was identified between the severity of RLS symptoms and diminished sleep quality (*r* = 0.571, *p* < 0.001). Children affected by both migraine and RLS were found to experience significantly poorer sleep quality in comparison to those with migraine alone. Among healthy controls, reduced sleep quality was also reported in individuals with RLS relative to their RLS-free counterparts, although the extent of impairment was less pronounced than that observed in the migraine group.

In line with previous evidence by Chen et al. (2010), who observed that migraine patients tend to experience more pronounced sleep difficulties than those with tension-type or cluster headaches, the present research also revealed a greater deterioration in sleep quality among children diagnosed with migraine (Chen et al., 2010). Notably, children with both migraine and RLS exhibited significantly higher PSQI scores than those with migraine alone or with other primary headache subtypes, while no significant variation in PSQI scores was detected within the TTH group. These observations suggest that poor sleep quality is a common feature across different types of primary headaches, yet the degree of impairment appears to be more substantial in migraine, particularly when RLS is present. Collectively, the findings suggest a possible association between sleep disturbance and headache; however, the direction and temporal sequence of this relationship cannot be determined based on the present data from a cross-sectional study.

The association between RLS severity and reduced sleep quality observed in this study has been consistently supported by prior research. For instance, Van Oosterhout et al. (2016) reported that individuals with co-occurring migraine and RLS exhibited significantly elevated PSQI scores compared to those with migraine alone, with symptom severity positively correlated with sleep disturbances (Van Oosterhout et al., 2016). The same study also revealed that pediatric migraine patients with RLS experienced significantly poorer sleep quality than healthy controls (*p* = 0.045), further highlighting the adverse impact of RLS symptoms on sleep. Supporting these findings, DelRosso, Bruni & Ferri (2020), through polysomnographic assessment, identified increased sympathetic nervous system activity in children with RLS during both pre-sleep and sleep periods, which was associated with disrupted sleep architecture (DelRosso, Bruni & Ferri, 2020). Expanding upon this evidence, Wang et al. (2019), in a comprehensive meta-analysis, emphasized the complex and multidimensional relationship among migraine, RLS, and sleep disturbances, suggesting the potential involvement of shared neurobiological mechanisms (Wang et al., 2019). Beyond its established role in sleep disturbance, RLS may represent an additional clinical burden contributing to impaired sleep quality in children with primary headache disorders. Its comorbidity appears to aggravate the clinical burden associated with primary headaches, particularly migraine. Accordingly, the early detection of RLS and the systematic evaluation of sleep quality are recommended as integral components of the clinical assessment for children presenting with recurrent headache complaints.

Several limitations should be acknowledged in the interpretation of these findings. First, the single-center design of the study inherently limits the generalizability of the results to broader pediatric populations. In addition, the exclusive use of self-reported questionnaires to assess sleep quality represents a methodological limitation, as such instruments are subject to recall and reporting biases and may not accurately capture objective sleep parameters. Another limitation is that the timing of RLS onset in children with primary headache disorders was not determined. The diagnosis of RLS was made during the study evaluation rather than based on prior clinical history, so it is unclear whether RLS develops before or after the onset of headache. Future longitudinal studies are needed to clarify this temporal relationship. Finally, the lack of distinction between migraine subtypes (with or without aura) restricted the possibility of conducting phenotype-specific analyses, thereby limiting the granularity and depth of interpretation.

## Conclusions

In this study, RLS was found to be significantly more prevalent and more severe in children with primary headache disorders, particularly those with migraine, compared to healthy controls. A strong positive correlation was observed between RLS symptom severity and impaired sleep, indicating a potential interaction between these conditions in pediatric populations. Beyond descriptive associations, mediation analysis indicated that the presence of RLS accounted for a part of the relationship between migraine and impaired sleep quality, while migraine also remained independently associated with sleep disturbance. Together, these findings expand the current understanding of headache–RLS comorbidity and its impact on sleep in children. However, the generalizability and objectivity of the results may be limited by the single-center study design and the exclusive use of self-reported sleep questionnaires. Future research should incorporate longitudinal approaches and employ objective measures, such as polysomnography, to better elucidate the directionality and underlying mechanisms of this association.

##  Supplemental Information

10.7717/peerj.20925/supp-1 Supplemental Information 1 STROBE Checklist

10.7717/peerj.20925/supp-2 Supplemental Information 2 Raw Dataset of the Study

## Additional Information and Declarations

### Data Availability

The following information was supplied regarding data availability: The raw data are available in the Supplemental File.

### Clinical Trial Registration

The following information was supplied regarding Clinical Trial registration: 118734
